# Prognostic Role of Pan-Immune-Inflammatory Value in Patients with Non-ST-Segment Elevation Acute Coronary Syndrome

**DOI:** 10.3390/jcdd12020079

**Published:** 2025-02-18

**Authors:** Jeong Tae Byoun, Kyeong Ho Yun, Sungho Jo, Donghyeon Joo, Jae Young Cho

**Affiliations:** Departments of Cardiovascular Medicine, Regional Cardiocerebrovascular Center, Wonkwang University Hospital, Iksan 54538, Republic of Korea; overchaos12@gmail.com (J.T.B.); fuenrostu@naver.com (S.J.); dhjoozz@naver.com (D.J.); librato46@gmail.com (J.Y.C.)

**Keywords:** acute coronary syndrome, biomarkers, neutrophils, lymphocytes, platelets

## Abstract

Blood cell-derived indices are potential predictors of clinical outcomes in coronary artery disease. This study assessed the prognostic value of the pan-immune-inflammatory value (PIV) for predicting 1-year major adverse cardiovascular events (MACEs) in patients with non-ST-segment elevation acute coronary syndrome (ACS). A retrospective cohort of 1651 patients receiving percutaneous coronary intervention was analyzed. PIV, calculated from blood cell counts, was categorized with a cut-off value of 256.3 (sensitivity 60.7%, specificity 59.3%) based on receiver operating characteristic curve analysis. MACEs were operationalized as a composite of all-cause mortality, myocardial infarction (MI), stroke, any revascularization, and rehospitalization for heart failure. The incidence of MACEs was 5.0% in patients with low PIV and 9.7% in those with high PIV (log-rank *p* < 0.001). Multivariate analysis identified age 65 > years, renal dysfunction (eGFR < 60 mL/min/1.73 m^2^), and high PIV (>256.3) (HR 1.49, 95% CI 1.01–2.22, *p* = 0.048) as independent predictors of MACEs. Subgroup analyses revealed no statistically significant interaction between MI status or C-reactive protein levels and PIV. PIV was an independent predictor of 1-year MACEs in patients with non-ST-segment elevation ACS. It may serve as a reliable prognostic marker independently of MI or C-reactive protein levels.

## 1. Introduction

Acute coronary syndrome (ACS) is a broad clinical entity encompassing conditions ranging from unstable angina to myocardial infarction (MI) [1]. In South Korea, the incidence of non-ST-elevation ACS is increasing, with an in-hospital mortality rate of about 3–4% [2]. However, the long-term mortality rate reached approximately 11% over 3 years, which was higher than that of ST-elevation myocardial infarction (MI) [3]. Early diagnosis and risk stratification are therefore critical for optimal management of ACS patients. Various clinical risk scores and biomarkers have been studied in this context. Blood cell-derived indices are inexpensive and readily applicable markers.

The inflammatory response plays a crucial role in the development, progression, and destabilization of atherosclerosis [4,5]. Atherosclerotic plaque rupture results from a complex interplay between innate and adaptive immunity. White blood cells and their subtypes reflect inflammatory and immune processes. Neutrophils, the first responders, mediate the inflammatory response to acute myocardial injury, leading to additional tissue damage and microcirculatory impairment [6]. Monocytes not only participate in atherosclerotic plaque development but also trigger pathological inflammatory processes during the early stages of myocardial injury. Upon transforming into macrophages, they initiate a cascade of myocardial inflammation following reperfusion in pathological states [7,8]. Lymphocytes, in contrast, play a distinct role compared to neutrophils and monocytes, reflecting a regulated, controlled inflammatory process that contributes to plaque stability [9,10]. Additionally, platelets play a critical role in atherosclerosis, with their activation promoting inflammation and thrombosis [11,12]. The four blood cell counts mentioned above have been widely studied as markers due to their low cost and ease of accessibility. In recent years, composite inflammatory indicators have been evaluated for providing a more comprehensive reflection of the inflammatory state.

Among these, the neutrophil-to-lymphocyte ratio (NLR) and platelet-to-lymphocyte ratio (PLR) are the most extensively studied markers for predicting clinical outcomes in patients with a wide range of cardiovascular diseases [13,14,15,16,17]. More recently, the systemic immune-inflammation index (SII), systemic inflammation response index (SIRI), and pan-immune-inflammatory value (PIV) have emerged as promising prognostic markers in coronary artery disease [18,19,20].

Among these indices, PIV is a relatively novel marker that has not been extensively studied in ACS patients. PIV is calculated based on the counts of neutrophils, monocytes, lymphocytes, and platelets, and thus theoretically reflects all systemic inflammation and immunity. In this study, we evaluated the prognostic role of PIV in ACS patients and compared its performance with other blood cell-derived indices. Specifically, we aimed to investigate the association between PIV and 1-year major adverse cardiovascular events (MACEs) in patients with unstable angina and non-ST-segment elevation MI.

## 2. Materials and Methods

### 2.1. Study Population

**Figure 1:**
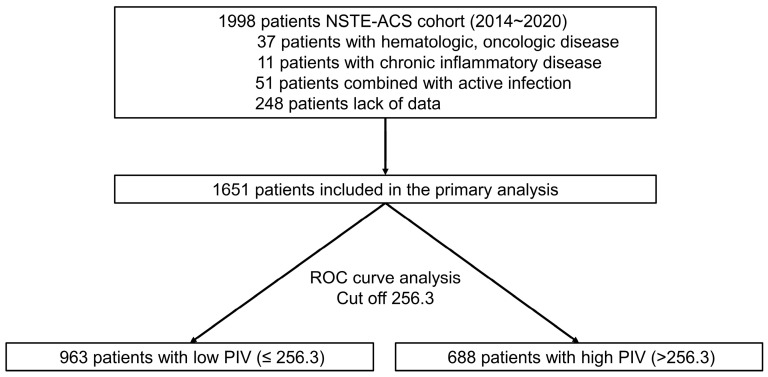
Participant flow in the present study.

From 2014 to 2020, we analyzed a single-center, retrospective cohort of 1998 non-ST-segment ACS patients who underwent percutaneous coronary intervention (PCI). The inclusion criteria were patients aged over 18 years with a life expectancy of more than 1 year. The exclusion criteria included patients with hematological, oncological, or chronic inflammatory diseases, those with active infections, and those with incomplete data. A flowchart illustrating the patient selection process is shown in Figure 1. Ultimately, 1651 patients were included in the study and followed throughout their clinical course. Data collection included patients’ baseline characteristics, PCI details, laboratory results, and 1-year clinical outcomes. The study protocol, which involved processing anonymized patient data, was approved by the Institutional Review Board (IRB) of Wonkwang University Hospital (2024-05-020, approval date 27 May 2024). Informed consent was waived by the IRB due to the retrospective and observational nature of the study, as well as the absence of any patient-identifying information.

### 2.2. Data Collection

Demographic data and risk factors, including age, gender, history of hypertension, diabetes, and current smoking, were collected from hospital records. Venous blood samples, routinely measured for blood urea nitrogen, creatinine, total cholesterol, triglycerides, high-density lipoprotein cholesterol, low-density lipoprotein cholesterol, baseline and peak troponin T levels, C-reactive protein, hemoglobin, neutrophil count, lymphocyte count, monocyte count, and platelet count, were recorded as well. All blood samples were collected at the time of hospital admission, prior to PCI. The glomerular filtration rate (eGFR) was calculated using the Modification of Diet in Renal Disease equation [21].

PIV was calculated as ((neutrophil count × platelet count × monocyte count)/lymphocyte count). SII was calculated as ((neutrophil count × platelet count)/lymphocyte count). SIRI was calculated as ((neutrophil count × monocyte count)/lymphocyte count).

### 2.3. Endpoint

The primary endpoint was 1-year MACEs, operationalized as a composite of all-cause death, MI, stroke, any revascularization, and rehospitalization for heart failure. Rehospitalization for heart failure was defined as an unplanned hospital admission following initial discharge, due to symptoms and signs of heart failure. The secondary endpoint included each individual component of MACEs during the 1-year follow-up.

### 2.4. Statistical Analyses

All analyses were conducted using SPSS version 27.0 (IBM Corp., Armonk, NY, USA). Continuous data are presented as means and standard deviations and were compared using the independent *t*-test. Categorical variables are presented as counts (percentages) and were compared using the chi-squared test or Fisher’s exact test.

Patients were stratified based on receiver operating characteristic (ROC) curve analysis. The optimal cut-off value for PIV in predicting MACEs was 256.3 (sensitivity: 60.7%, specificity: 59.3%). A Cox proportional hazard model was used to assess whether high PIV contributed to MACEs, with results reported as hazard ratios (HRs), adjusted for age and gender, along with corresponding 95% confidence intervals (CIs). The Kaplan–Meier method with the log-rank test was used to determine the cumulative incidence of MACEs. If patients experienced multiple components of the composite primary outcome, they were assessed until the first event occurred. A multivariate Cox proportional hazard model was constructed to predict MACEs. Variables with a *p*-value < 0.05 in the univariate analysis were included in the multivariate model to examine the comprehensive effects of PIV on the endpoint. A mixed-effect model was used to identify random effects for the primary outcome in subgroup analyses. The heterogeneity of effects across subgroups was assessed by introducing interaction terms in the Cox proportional hazard model. ROC curve analyses of NLR, PLR, SII, and SIRI were performed, and cut-off points were determined using Youden’s index. All *p*-values were two-sided, and statistical significance was set at *p* < 0.05.

## 3. Results

Baseline clinical and angiographic characteristics are shown in Table 1. Patients with high PIV exhibited worse clinical characteristics, including a higher incidence of hypertension, diabetes, smoking, and MI. Patients with high PIV also had higher neutrophil, monocyte, and platelet counts, as well as lower hemoglobin and eGFR levels compared to those with low PIV. However, the two groups showed similar angiographic and procedural characteristics.

**Figure 2:**
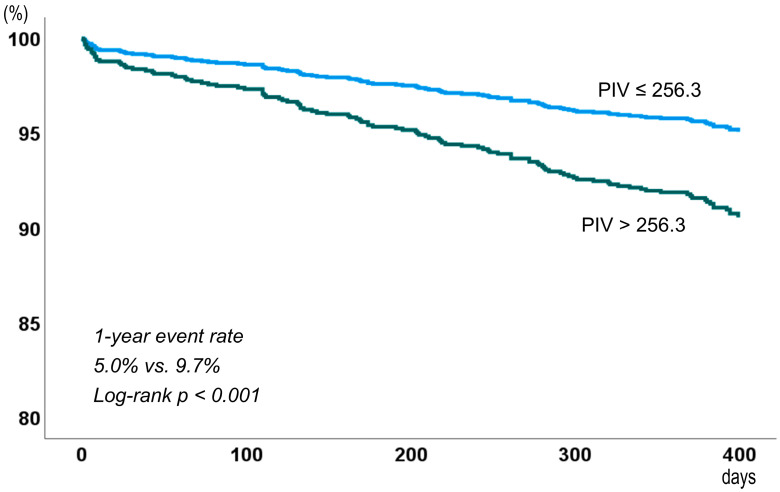
Event-free survival curve based on the pan-immune-inflammatory value (PIV).

MACEs occurred in 6.9% of patients (111/1606) during the 1-year follow-up (median 400 days, interquartile range 367.8–400 days). The incidence of MACEs was 5.0% in patients with low PIV and 9.7% in those with high PIV (log-rank *p* < 0.001) (Figure 2). As shown in Table 2, the incidence of all-cause death, cardiac death, and stroke was significantly higher in patients with high PIV than in those with low PIV.

**Figure 3:**
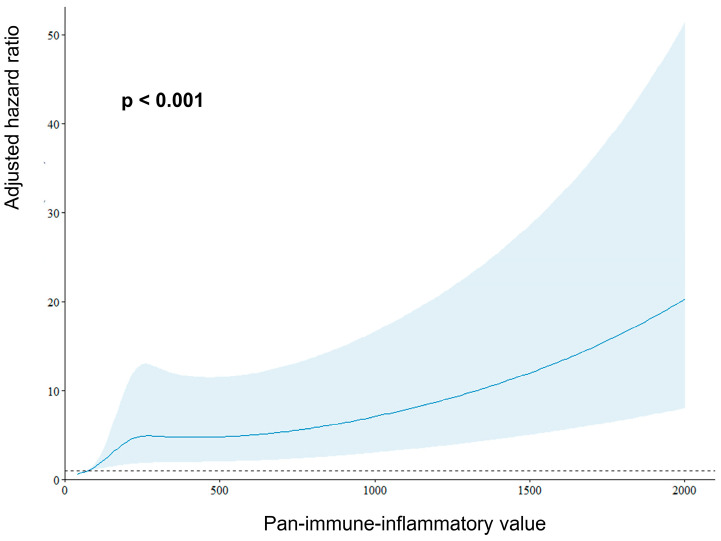
Prognostic impact of pan-immune-inflammatory value as a continuous variable on the risk of 1-year major adverse cardiovascular events. A restricted cubic spline curve was drawn to demonstrate the continuous prognostic effect of PIV on the risk of 1-year MACEs in patients with non-ST-segment elevation acute coronary syndrome. Adjusted variables were age, gender, hypertension, diabetes, hemoglobin, eGFR, C-reactive protein, ejection fraction, and troponin T.

Multivariate analysis revealed that age 65 > years (adjusted HR 1.75, 95% CI 1.12–2.72, *p* = 0.014), renal dysfunction (eGFR < 60 mL/min/1.73 m^2^) (adjusted HR 1.73, 95% CI 1.23–2.64, *p* = 0.012), and high PIV (>256.3) (adjusted HR 1.49, 95% CI 1.01–2.22, *p* = 0.048) were independent predictors of MACEs (Table 3). There was a significant association between the PIV and risk of MACEs as a continuous value (*p* < 0.001, Figure 3).

To investigate whether other variables affect the relationship between PIV and MACEs, additional subgroup analyses were conducted (Table 4). Subgroup analyses revealed no statistically significant interactions, except for age (*p* < 0.001 for interaction), history of hypertension (*p* < 0.001 for interaction), and eGFR (*p* = 0.003 for interaction).

ROC curve analyses were performed to compare the predictive performance of PIV with other inflammatory indices, including NLR, PLR, SII, and SIRI (Table 5). ROC curve analysis suggested that the optimal PIV cut-off value was 256.3, with PIV > 256.3 predicting 1-year MACEs in ACS patients with a sensitivity of 60.7% and specificity of 59.3%. The area under the curve (AUC) for PIV was higher than that for the other indices.

## 4. Discussion

The main findings of this study were: (1) PIV is an independent predictor of 1-year MACEs in non-ST-segment elevation ACS patients; (2) PIV is a potential marker for predicting MACEs, regardless of MI or C-reactive protein levels; and (3) PIV appears superior to other blood cell-derived indices in predicting MACEs in patients with unstable angina and non-ST-segment elevation MI. To the best of our knowledge, this is the first study to evaluate the prognostic role of PIV in predicting MACEs in non-ST-segment elevation ACS.

In recent years, composite inflammatory markers have been evaluated to more comprehensively reflect the inflammatory state. Several cohort studies and meta-analyses have shown that a high NLR is associated with an increased risk of all-cause mortality and cardiovascular events in patients with ST-segment elevation MI and those undergoing cardiac revascularization [14,22]. PLR is also strongly associated with all-cause mortality and MACEs in MI patients, both during hospitalization and in long-term follow-up [15,23]. The combination of neutrophil, lymphocyte, and platelet counts led to the development of SII. SII is an inflammation-related marker that integrates neutrophil, platelet, and lymphocyte counts, reflecting the overall immune-inflammatory state in the body [24]. SII has been shown to be a useful predictor of clinical outcomes in cancer, MI, and other inflammatory diseases [18,25]. Similarly, the SIRI, which integrates neutrophil, monocyte, and lymphocyte counts, indicates a significant pro-inflammatory reaction mediated by monocytes and neutrophils, alongside a reduced anti-inflammatory effect mediated by lymphocytes. In ACS patients undergoing PCI, SIRI has been found to be a strong and independent risk factor for MACEs and rehospitalization for heart failure [26].

Recently, a new-parameter PIV, including all blood cell populations reflecting systemic inflammation and immunity, was proposed as a more robust and reliable indicator for predicting clinical outcomes in patients with advanced colorectal cancer [27]. Subsequently, the predictive significance of PIV was evaluated in MI patients. Murat et al. reported that high PIV (greater than 906.14) was associated with an increased 1-year all-cause mortality rate in 658 ST-segment elevation MI patients [20]. Yang et al. found that PIV was correlated with the severity of coronary stenosis and in-hospital MACEs, including death, MI, stroke, and new heart failure, in 542 ST-segment elevation MI patients [28]. More recently, in patients with non-ST-segment elevation MI, PIV was significantly correlated with a high SYNTAX score and the severity of coronary artery disease [29]. However, the prognostic efficacy of PIV in patients with non-ST-segment elevation MI following PCI has not yet been investigated.

Our study is the first to show that PIV is associated with 1-year MACEs in ACS patients. Patients with higher PIV had worse clinical characteristics: higher incidence of risk factors, lower ejection fraction and renal function, and higher C-reactive protein levels. Nevertheless, after adjusting for covariates, higher PIV was an independent predictor of MACEs. Therefore, PIV is considered to be a good predictor for MACEs that is cheap and easy to apply. However, it remains questionable whether PIV is significantly superior to other blood cell-derived indices. Although the AUC for PIV is larger than for other indices, the difference is not substantial, and similar findings have been reported in other studies. Additionally, the AUC was less than 0.7, indicating that the ROC model for blood cell-derived indices has a poor correlation. Therefore, the cut-off point is not meaningful for selecting patients at risk of MACEs, but should instead be viewed as a continuous variable indicating poor prognosis. Another important aspect of this study is the extension of the significance of PIV to patients without MI. While previous studies have mainly focused on the effects of blood cell-derived indices in patients with ST-segment elevation MI, this study demonstrates that PIV is a useful prognostic indicator even in patients with unstable angina. Moreover, PIV appears to predict MACEs regardless of C-reactive protein levels. Traditionally, C-reactive protein has been well established as an inflammatory marker, but in our study, even in patients with low C-reactive protein levels, PIV was still associated with 1-year MACEs. Further research is needed to confirm and expand these findings in larger, more varied patient populations.

This study has several limitations. First, the single-center, retrospective design introduces the potential for bias, which may limit the generalizability of the results. Future research will aim to include larger samples and patients from multiple regions. Second, C-reactive protein and troponin were not measured using sensitivity methods.

## 5. Conclusions

In conclusion, PIV is an independent predictor of 1-year MACEs in non-ST-segment elevation ACS patients. It may serve as a potential predictor of MACEs independently of MI or C-reactive protein levels.

## Figures and Tables

**Table 1:** Baseline clinical characteristics.

	PIV ≤ 256.3 (*n* = 963)	PIV > 256.3 (*n* = 688)	*p*-Value
Age (years)	65.9 ± 10.3	66.9 ± 11.5	0.082
Male (%)	656 (68.1)	456 (66.3)	0.431
Body mass index (kg/m^2^)	24.9 ± 3.0	24.7 ± 3.7	0.450
Hypertension (%)	605 (62.8)	486 (70.6)	0.001
Diabetes mellitus (%)	370 (38.4)	342 (49.7)	<0.001
Current smoker (%)	234 (24.3)	215 (31.3)	0.002
Previous coronary intervention (%)	95 (9.9)	75 (10.9)	0.495
Diagnosis (%)			<0.001
Unstable angina	841 (87.3)	495 (71.9)	
Non-ST-segment elevation MI	122 (12.7)	193 (28.1)	
Ejection fraction (%)	60.2 ± 11.1	55.4 ± 13.0	<0.001
Hemoglobin (g/dL)	13.6 ± 1.8	13.2 ± 2.1	<0.001
Neutrophil count (10^3^/μL)	3.6 ± 1.1	6.6 ± 2.6	<0.001
Lymphocyte count (10^3^/μL)	2.4 ± 0.9	2.1 ± 0.8	<0.001
Monocyte count (10^3^/μL)	0.5 ± 0.1	0.7 ± 0.2	<0.001
Platelet count (10^3^/μL)	211.0 ± 49.2	250.4 ± 68.7	<0.001
eGFR (mL/min/1.73 m^2^)	77.5 ± 23.0	73.0 ± 31.3	0.001
Troponin T (ng/mL)	0.23 ± 0.76	0.44 ± 1.02	<0.001
LDL cholesterol (mg/dL)	106.4 ± 40.3	106.4 ± 41.7	0.992
C-reactive protein (mg/L)	2.8 ± 5.0	9.9 ± 22.3	<0.001
Culprit lesion (%)			0.189
Left main	52 (5.4)	29 (4.2)	
Left anterior descending	479 (49.7)	318 (46.2)	
Left circumflex	189 (19.6)	139 (20.2)	
Right coronary artery	243 (25.2)	202 (29.4)	
Multivessel disease (%)	447 (46.4)	341 (49.6)	0.207
Stent number per patient	1.6 ± 0.9	1.6 ± 1.0	0.714
Minimal stent diameter (mm)	2.9 ± 0.4	2.9 ± 0.4	0.534
Total stent length (mm)	43.3 ± 27.7	44.7 ± 29.6	0.320
Discharge medication (%)			
Aspirin	961 (99.8)	688 (100)	0.514
P2Y12 inhibitors	951 (98.8)	674 (98.0)	0.204
Beta blocker	606 (62.9)	454 (66.0)	0.201
ACEI/ARB	826 (85.8)	603 (87.6)	0.272
Statin	941 (97.7)	673 (97.8)	0.888

PIV, pan-immune-inflammatory value; MI, myocardial infarction; eGFR, estimated glomerular filtration rate; LDL, low-density lipoprotein; ACEI, angiotensin-converting enzyme inhibitor; ARB, angiotensin receptor blocker.

**Table 2:** Clinical outcomes based on pan-immune-inflammatory value.

	PIV ≤ 256.3 (*n* = 963)	PIV > 256.3 (*n* = 688)	Adjusted HR (95% CI)	*p*-Value
All-cause death	9 (0.9)	27 (3.9)	4.03 (1.89, 8.57)	<0.001
Cardiac death	7 (0.7)	17 (2.5)	3.24 (1.34, 7.81)	0.009
Myocardial infarction	16 (1.7)	13 (1.9)	1.14 (0.55, 2.37)	0.725
Stroke	5 (0.5)	12 (1.7)	3.30 (1.16, 9.36)	0.025
Any revascularization	26 (2.7)	17 (2.5)	0.92 (0.50, 1.70)	0.799
Rehospitalization for heart failure	9 (0.9)	12 (1.7)	1.85 (0.78, 4.40)	0.162
Major adverse cardiovascular events	48 (5.0)	67 (9.7)	1.96 (1.36, 2.85)	<0.001

PIV, pan-immune-inflammatory value; HR, hazard ratio; CI, confidence interval.

**Table 3:** Predictors of 1-year major adverse cardiovascular events.

	Univariate Analysis		Multivariate Analysis	
	HR (95% CI)	*p*-Value	Adjusted HR (95% CI)	*p*-Value
Age > 65 years	2.29 (1.50, 3.48)	<0.001	1.75 (1.12, 2.72)	0.014
Male gender	1.11 (0.76, 1.63)	0.591		
Hypertension	1.50 (1.19, 1.90)	0.001	1.41 (0.86, 2.31)	0.171
Diabetes mellitus	1.38 (1.15, 1.66)	0.001	1.47 (0.99, 2.18)	0.057
Myocardial infarction	1.62 (1.08, 2.45)	0.021	1.37 (0.90, 2.10)	0.146
Hemoglobin < 12 g/dL	2.43 (1.67, 3.55)	<0.001	1.34 (0.88, 2.05)	0.178
eGFR < 60 mL/min/1.73 m^2^	2.69 (1.86, 3.89)	<0.001	1.73 (1.23, 2.64)	0.012
C-reactive protein > 2 mg/L	1.75 (1.20, 2.54)	0.003	1.32 (0.89, 1.95)	0.171
Ejection fraction < 50%	1.25 (1.03, 1.53)	0.024	1.05 (0.69, 1.61)	0.817
PIV > 256.3	2.01 (1.39, 2.91)	<0.001	1.49 (1.01, 2.22)	0.048

HR, hazard ratio; CI, confidence interval; eGFR, estimated glomerular filtration rate; PIV, pan-immune-inflammatory value.

**Table 4:** Subgroup analysis for major adverse cardiovascular events.

Subgroup	PIV ≤ 256.3	PIV > 256.3	HR (95% CI)	*p*-Value	*p*-Value for Interaction
Age, years					<0.001
<65	18/428 (4.2)	11/273 (4.0)	0.95 (0.45, 2.01)	0.895	
≥65	30/535 (5.6)	58/415 (13.5)	2.54 (1.63, 3.95)	<0.001	
Gender					0.885
Male	34/656 (5.2)	41/456 (9.0)	1.76 (1.12, 2.78)	0.015	
Female	14/307 (4.6)	26/232 (11.2)	2.60 (1.36, 4.97)	0.004	
Hypertension					<0.001
Yes	35/605 (5.8)	58/486 (11.9)	2.12 (1.40, 3.23)	<0.001	
No	13/358 (3.6)	9/202 (4.5)	1.25 (0.54, 2.93)	0.605	
Diabetes					0.077
Yes	28/370 (7.6)	39/342 (11.4)	1.55 (0.95, 2.52)	0.077	
No	20/593 (3.4)	28/346 (8.1)	2.45 (1.38, 4.36)	0.002	
Clinical presentation					0.317
Unstable angina	43/841 (5.1)	41/495 (8.3)	1.65 (1.08, 2.53)	0.022	
Non-ST-segment elevation MI	5/122 (4.1)	26/196 (13.5)	3.44 (1.32, 8.96)	0.011	
Multivessel disease					0.050
Yes	28/447 (6.8)	41/341 (12.0)	1.99 (1.23, 3.22)	0.005	
No	20/516 (3.9)	26/347 (7.5)	1.97 (1.10, 3.54)	0.022	
Hemoglobin, g/dL					0.073
<12	15/168 (8.9)	28/175 (16.0)	1.95 (1.04, 3.65)	0.037	
≥12	33/795 (4.2)	39/513 (7.6)	1.85 (1.16, 2.94)	0.009	
eGFR, mL/min/1.73 m^2^					0.003
<60	15/178 (8.4)	35/213 (16.4)	2.03 (1.11, 3.71)	0.022	
≥60	33/785 (4.2)	32/475 (6.7)	1.62 (0.99, 2.64)	0.051	
C-reactive protein, mg/L					0.052
≤2.0	27/645 (4.2)	25/313 (8.0)	1.94 (1.12, 3.33)	0.017	
>2.0	19/286 (6.6)	40/362 (11.0)	1.72 (1.00, 2.97)	0.052	

PIV, pan-immune-inflammatory value; MI, myocardial infarction; eGFR, estimated glomerular filtration rate.

**Table 5:** Receiver operating characteristic curve analysis of blood cell derived markers.

Variables	Area Under Curve	*p*-Value	Cut Off	Sensitivity	Specificity
Pan-immune-inflammatory value	0.658	<0.001	256.3	60.7%	59.3%
Neutrophil-to-lymphocyte ratio	0.623	<0.001	3.06	42.7%	79.6%
Platelet-to-lymphocyte ratio	0.633	<0.001	83.2	89.6%	30.7%
Systemic immune-inflammation index	0.635	<0.001	752.7	39.1%	81.4%
Systemic inflammation response index	0.652	<0.001	1.43	52.2%	70.6%

## Data Availability

The datasets presented in this article are not readily available due to ethical restrictions. Requests for access to the datasets should be directed to the corresponding author.
